# Long term clinical performance of 10 871 dental implants with up to 22 years of follow‐up: A cohort study in 4247 patients

**DOI:** 10.1111/cid.12994

**Published:** 2021-03-25

**Authors:** David French, Ronen Ofec, Liran Levin

**Affiliations:** ^1^ Department of Periodontics, Faculty of Dentistry University of British Columbia Vancouver Canada; ^2^ Private Practice Calgary Canada; ^3^ Department of Statistics and Operations Research Tel‐Aviv University Tel‐Aviv Israel; ^4^ Private Dental Practice Tel‐Aviv Israel; ^5^ Faculty of Medicine and Dentistry University of Alberta Alberta Canada

**Keywords:** augmentation, bone loss, mucositis, peri‐implantitis, success, survival

## Abstract

**Background:**

The present retrospective study was aimed to assess the long‐term clinical performance of dental implants in a cohort study of 4247 patients.

**Methods:**

A longitudinal observational cohort study was done on all implants performed by a single periodontist from July 1995 to April 2019. The main outcome variables of this study were implant failure and marginal bone level around implants.

**Results:**

The study participants received a total of 10 871 implants with a mean of 2.56 implants per patient. The cohort was followed‐up to 22.2 years (mean = 4.5 ± 4.2). Among the 4247 patients of the current study, 140 patients (3.3%) experienced a combined total of 178 implant failures. According to life table analysis, at the implant level the cumulative survival rate at 3, 5, 10, and 15 years was 98.9%, 98.5%, 96.8%, and 94.0%, respectively while at patient level was 97.4%, 96.7%, 92.5%, and 86% at 3, 5, 10, and 15 years. Patients with multiple units were at a greater risk for having an implant failure. Baseline bone level was 0.09 ± 0.28 mm while at 8–10 years the mean bone level was 0.49 ± 0.74 mm. The incidence of peri‐implant mucositis at the implant level was 9.4% at 2–3 years, 9.3% at 4–5 years, 12.1% at 6–7 years, and 11.9% at 8–10 years. The incidence of peri‐implantitis was 2%, 2.6%, 3.2%, and 7.1% at 2–3, 4–5, 6–7, and 8–10 years, respectively. Cigarette smoking and diabetes mellitus were positively correlated with implant failure.

**Conclusions:**

Though the results are promising and encouraging in terms of survival and bone level over time, it is important to emphasize the potential risk factors and consider them prior to dental implant placement.

**What is known:**

- Dental implants are known to have high survival and success rates.
- Long‐term, large‐scale, “real life” follow‐ups and documentation are needed to better understand the behavior of dental implants over time as well as the factors influencing the survival and success of dental implants.

**What this study adds:**

- This long‐term, large‐scale, “real life” retrospective analysis provides a statistical analysis of factors related to dental implants' survival and success.
- Though the results are promising and encouraging in terms of survival and bone level over time, it is important to emphasize the potential risk factors and consider them prior to dental implant placement. It is of utmost importance to highlight the role of proper preparation and maintenance for the long‐term outcomes.

## 1 INTRODUCTION

Edentulism is a serious health problem involving functional, esthetic, phonetic, and psychological problems.^1^, ^2^ Despite great achievements in global oral health, edentulism remains a major and irreversible problem affecting the quality of life.

Globally, it was reported in 2017 that there were 3.5 billion cases of oral conditions, of which 2.3 billion had untreated caries in permanent teeth, 796 million had severe periodontitis and 267 million had total tooth loss.^3^

In providing adequate therapy options for these patients, information regarding long‐term outcomes plays a key role in the decision‐making process. Dental implants can pose a viable solution for partially and fully edentulous patients.^4^ The success of implant rehabilitation relies on the integration of the implants in hard and soft tissues. Marginal bone loss (MBL) is, therefore, a critical factor affecting the clinical outcome.^1^, ^4^ Multifactorial reasons for implant failure and MBL are reported in the literature, however, they are not all fully understood.^4^, ^5^, ^6^, ^7^

Despite the high survival rate in many studies, implant‐supported prostheses are not free from complications and morbidity, and their longevity is limited not only by biologic complications but also by prosthetic maintenance requirements and the restoration issues.^8^, ^9^, ^10^ Implant complications and failures lengthen and complicate the treatment process, as well as jeopardize the clinician's efforts to accomplish satisfactory function and esthetics. For the patient, this usually involves further cost and additional procedures.

Criteria and data for implant success should serve as an aid for clinical follow‐up and to help evaluate the clinical outcomes of different implant systems in research. Long‐term collection and analysis of data are of the utmost importance when evaluating a procedure such as dental implant placement. It should help the clinician assess a given condition and predict its future clinical course, as well as help in decision making with regards to additional therapy, frequency of follow‐up, and hygiene appointments.^10^, ^11^ Therefore, it should be of interest to investigate and continuously report on the outcome of the long‐term evaluation of dental implant performance in routine practice. In this context, the present retrospective study was aimed to assess the long‐term clinical performance of dental implants with up to 22 years of follow‐up in a cohort study of 4247 patients treated in a single dental office. The study design is a useful method to get insight into the clinical performance of dental implants in the large scale, realistic situation of private practice, as opposed to the small‐scale sterile situations frequently obtained from randomized clinical trials.

## 2 METHODS

This was a longitudinal observational cohort study reporting on 10 871 implants, performed by a single periodontist (DF) from July 1995 to April 2019. Restorations were performed by a variety of General Dentists and Specialists in the Calgary, Alberta region. All measurements were taken by the same examiner who placed the implants (DF). The inclusion criterion was partially or fully edentulous sites, and the only exclusion criterion was patients with ASA class 3 or higher. Implants were placed according to manufacturer guidelines. The location of implants was determined based on the individual patient's requirements. Patient consent was obtained, the study was approved by Clinical Research Ethics Board at the University of British Columbia (#H18‐00315) and was conducted in accordance with the Helsinki Declaration of 1975, as revised in 2013. Data collection and analysis was designed to ensure patients' anonymity.

Data regarding medical, and dental status before surgery were available for analysis. These parameters (e.g., smoking, diabetes mellitus) were considered as baseline factors. The investigated variables were grouped into: implant characteristics (e.g., length, diameter), surgical site (e.g., location, etc.), procedures (e.g., insertion torque, augmentation, etc.) and prosthetic variables. Dates of the following clinical events were recorded: implant placement, stage 2 (3 months after implant placement and prior to prosthetic connection) and all follow‐up visits including the last (most recent) date the patient was seen as well as implant removal where applicable. The main outcome variable of this study were implant failure and marginal bone level around implants. Failure at implant level was defined as the removal of an implant for any reason. Failure at patient level was defined as a patient that experienced at least one implant failure during the follow‐up period. Early failures were defined as failures occurring before implant loading, while late failures occurred after loading. Survival time was defined as the time from implant insertion to implant removal or to last follow‐up for surviving implants.

Bone level measurements around implants had been performed at stage 2 surgery, and in years 1, 2–3, 4–5, 6–7 and 8–10 after installation as previously described.^12^, ^13^, ^14^

Peri‐implant soft tissue was evaluated by probing with a light vertical probe force of 17 g using a calibrated force automated probe or manual probe calibrated to about 17 g; each with a probe tip width of 0.45 mm at six locations around the implant. The soft tissue condition based on probing was determined using the implant mucosal index (IMI) whereby 0 = no bleeding, 1 = minimal single‐point bleeding, 2 = moderate multipoint bleeding, 3 = profuse multipoint bleeding, and 4 = suppuration.^14^, ^15^ Scores were applied to each implant as the worst point during entire implant follow‐up period. Peri‐implant mucositis was defined as IMI ≥ 2 not accompanied with bone loss whereas peri‐implantitis was defined as bone loss ≥1.0 mm in conjunction with IMI ≥ 2 at any follow‐up after the stage 2 baseline.

### 2.1 Statistical methods

Statistical analysis was performed at patient and implant level. Patient level analysis included all variables that describe the patient while implant level analysis included variables which describe a particular implant or the site/procedure around an implant. Although the diagnosis of mucositis, peri‐implantitis and failure are usually attributed to a particular implant, in the current study these outcome variables were also considered at patient level. In this context a “patient failure” means a patient who experienced at least one implant failure during the follow up period.

For continuous variables, the mean, mode and median were calculated in order to summarize the central tendency, while the standard deviation and range were calculated in order to estimate the dispersion. A 95% confidence interval was calculated in order to estimate the mean. Nominal and ordinal scale variables were presented in terms of frequency tables.

In order to describe the survival data, cumulative survival rate (CSR) was calculated according to the life table method and illustrated the results with the Kaplan–Meier survival curve. These methods calculate the number of patients at risk in each interval of time after excluding the censored observation from the analysis before the start of the interval.

Hazard ratios (HR) were calculated in order to estimate the association between explanatory variables and failure time. Hazard ratio for categorical variable is defined as the ratio between hazards for implant failure among one group compared to another group. A ratio equal to one means that hazards are equal across groups while HR < 1 and HR > 1 means protective and risk effect, respectively. Hazard ratios were obtained by constructing the Cox proportional hazard (PH) regression model. All explanatory variables of the current study were evaluated one by one in a univariate analysis. Variables that were significantly related to failure in a univariate analysis were incorporated into a multivariate model in order to account for confounding effect between certain variables. In our model we accounted for possible Intra cluster correlation (as a result of multiple implants within certain patients) by calculating sandwich type robust standard errors. Lastly, to use the Cox model, it was essential to check the underlying PH assumption, which states that HR is constant throughout the time under investigation. In the current analysis, the PH assumption was tested by using the Grambsch–Therneau test. In case of violation, we included a time‐variant covariate. Statistical analyses were performed with SPSS (IBM, Version 25.0, Armonk, NY) and R software (R Foundation for Statistical Computing, Vienna, Austria). All tests were two‐tailed with statistical significance level of 0.05 adopted.

## 3 RESULTS

**FIGURE 1:**
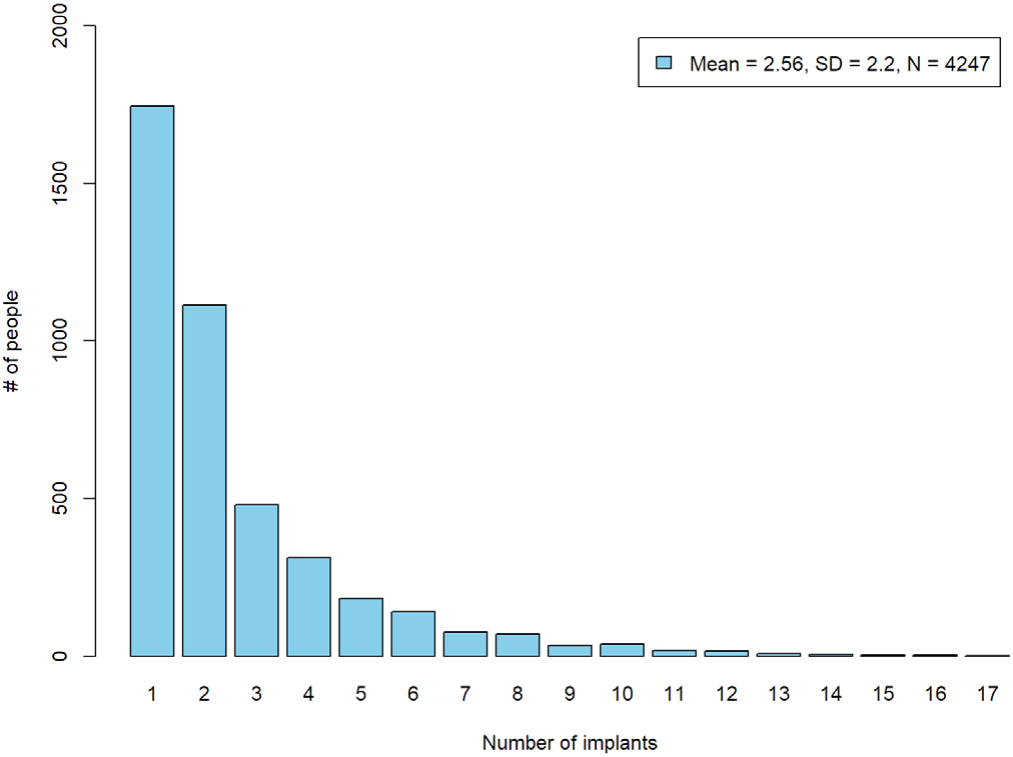
Number of implants per patient

**FIGURE 2:**
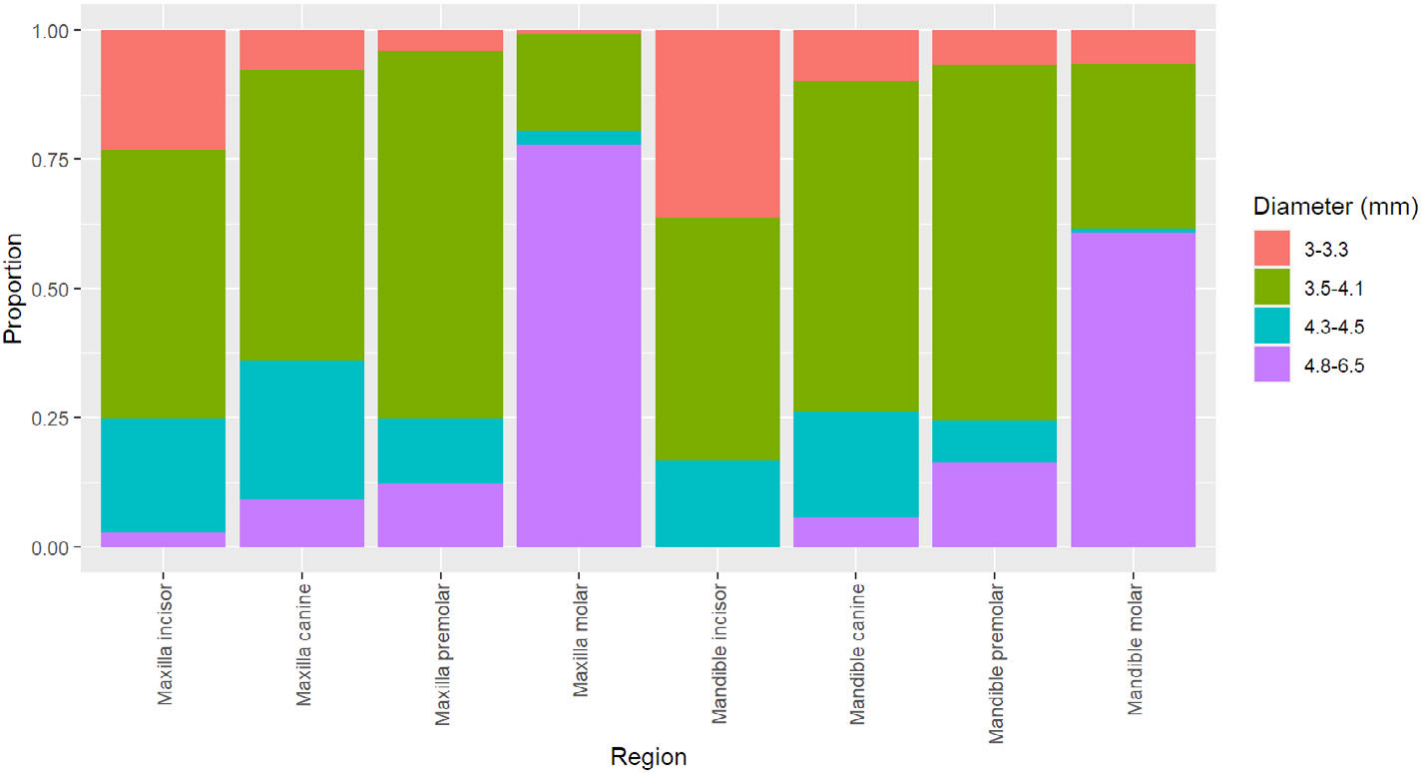
Conditional frequency distribution of implant diameter by location

Overall, the study cohort included 4247 patients (56.4% females) with a mean age at surgery equal to 53.8 ± 13.5 years. The study participants received a total of 10 871 implants (57.3% in the maxilla) with a mean number of implants per patient equal to 2.56, ranging from 1 to 17, and a mode (frequent) of 1 implant per patient (Figure 1). The most frequent (mode) implant location was the mandibular molar area (25.2%) followed by the maxillary incisor area (19.9%) and the maxillary premolar region (17.2%). The Straumann tissue level standard implant with an SLA surface was the dominant (55.2%) implant design and was used throughout the entire study period, as opposed to tapered implants with a limited use between 2005 and 2012 and Bone Level implants which started only from 2007. The most used implants by diameter/length were the 4.8/10, 4.1/10, and 4.1/12 (Figure 2). Narrow implants were dominant in the incisor area while wider implants were frequently used in the molars area. For the restorations, 34.5% of the implants were restored as a single unit crown, 47.9% as multiple unit splinted or bridged and 7% with removable bars or balls.

**FIGURE 3:**
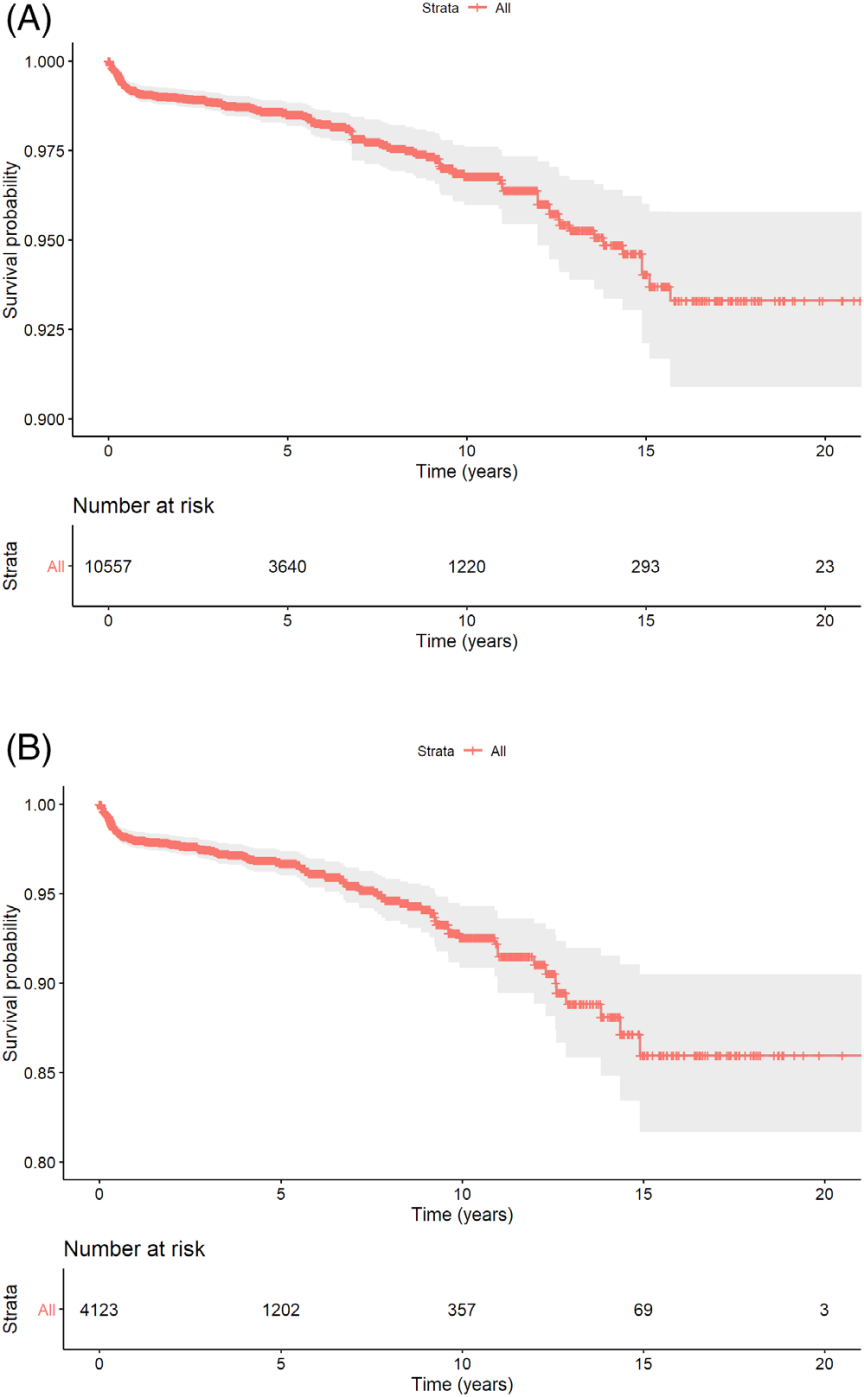
Kaplan–Meier survival curve (with robust standard errors). A, Implant level; B, patient level

The cohort was followed‐up up to 22.2 years, with a mean equal to 4.5 ± 4.2 years. Twenty‐five percent of the implants were followed‐up up to 17.6 months; however, 25% of implantations were performed after January 2014, with less chance for a long‐term follow‐up. At the implant level, 52 (0.5%) failures were observed during the surgical phase (before loading) and 126 (1.2%) failures were recorded after loading. The CSR at 3, 5, 10, and 15 years was 98.9%, 98.5%, 96.8%, and 94.0%, respectively (Figure 3A).

Among the 4247 patients of the current study, 140 patients (3.3%) experienced a combined total of 178 implant failures. According to life table analysis, the cumulative survival rate (CSR) at patient level was 97.4%, 96.7%, 92.5%, and 86% at 3, 5, 10, and 15 years (Figure 3B). From a patient perspective, patients with multiple implants were at a greater risk for experiencing a failure (10‐year CSR = 90.2%) compared to patients with a single unit (10‐year CSR = 98.2%).

Table 1 summarizes and compares the results for CSR's at patient and implant levels. As seen from Table 1 the results at the implant level are more optimistic compared to patient level analysis/.

**TABLE 1:** Cumulative survival rates at patient and implant level

Time (years)	Patient level		Implant level	
	CSR (%)	95% CI	CSR	95% CI
3	97.4	(96.9, 98.0)	98.9	(98.6, 99.1)
5	96.7	(96.0, 97.4)	98.5	(98.2, 98.8)
10	92.5	(90.8, 94.3)	96.8	(96.2, 97.4)
15	86.0	(81.7, 90.5)	94.0	(92.5, 95.6)

**FIGURE 4:**
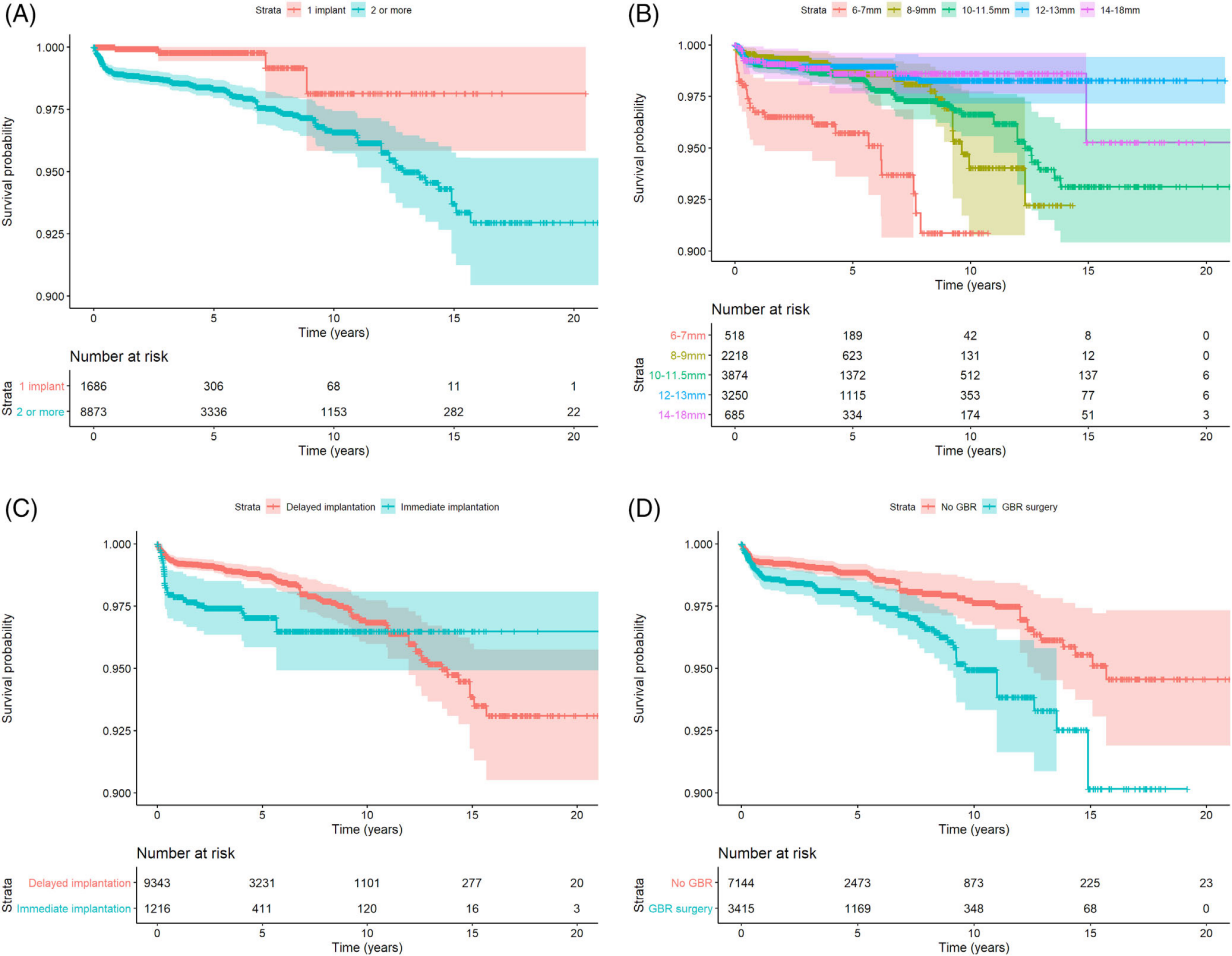
Survival curves (with robust standard errors) by (A) number of units; (B) implant length; (C) immediate implantation; (D) GBR surgery

Modeling of time until failure as an outcome variable revealed several significant associations. Table 2 presents the results of a univariate models for the study exploratory variables. Significant variables were incorporated into a multivariate model (Table 3). Hazard ratio (HR) for time until failure, when comparing implants in patients with multiple implants vs implants in patients who only have a single implant, was 5.85 (*p* < 0.00001; Figure 4A).

**TABLE 2:** Hazard ratios obtained from univariate Cox PH regressions

	Hazard ratio	95% CI	Robust *p*_value_	Violation of the PH assumption
Demographic variables				
Gender				
Male	1			
Female	0.83	(0.58, 1.18)	0.3	No
Age at 1st surgery (years)	0.99	(0.98, 1.01)	0.68	No
Health status variables				
Diabetes mellitus				
No	1			
Yes	2.17	(1.01, 4.68)	**0.048**	No
Periodontal disease				
No	1			
Yes	1.77	(0.89, 3.51)	0.10	No
Heavy smoking (>10 pack‐years)				
No	1			
Yes	2.02	(1.13, 3.59)	**0.017**	No
Implant specific variables				
Diameter (mm)	1.45	(0.99, 2.11)	0.051	0.04
Length (mm)	0.85	(0.78, 0.92)	<0.01	0.02
6 mm implant				
No	1			No
Yes	3.53	(2.25, 5.55)	**<0.01**	
Straumann design				
Standard (2.8 mm)	1		<0.01	
Standard Plus (1.8 mm)	1.77	(0.41, 7.64)	0.44	
Tapered Effect (1.8 mm)	2.90	(1.07, 7.89)	0.04	
Bone Level	0.74	(0.38, 1.43)	0.38	
Bone level taper	2.97	(0.70, 12.62)	0.14	
not Straumann	1.31	(0.85, 2.02)	0.22	
Multiple implants				
Single implant	1			
2 or more implants per patient	5.85	(2.14, 15.94)	**<0.01**	No
Surgical and site specific variables				
Jaw				
Mandible	1			
Maxilla	1.37	(0.96, 1.95)	0.09	<0.01
Location				
Anterior Mandible	1		0.01	
Posterior Mandible	0.87	(0.41, 1.84)	0.72	
Anterior Maxilla	1.15	(0.53, 2.47)	0.73	
Posterior Maxilla	1.27	(0.61, 2.65)	0.53	
Immediate implantation				
No	1			
Yes	1.78	(1.14, 2.78)	**0.01**	<0.01
Loading				
Delayed	1			
Immediate	1.14	(0.56, 2.31)	0.72	No
GBR surgery				
No	1			
Yes	1.85	(1.32, 2.62)	**<0.01**	No
Insertion torque (N cm)	0.98	(0.96, 1.01)	0.10	<0.01

**TABLE 3:** Hazard ratios obtained from multivariate Cox PH regression model

Variable	Hazard ratio	95% CI	*p*_value_
Multiple vs. single	5.19	(1.89, 14.34)	<0.01
Heavy smoking	1.81	(1.03, 3.17)	0.039
Diabetes mellitus	2.25	(1.04, 4.89)	0.040
Implant length: 6 mm	3.46	(2.22, 5.38)	<0.01
Immediate implantation	2.44	(1.53, 3.90)	<0.01^a^
GBR surgery	1.98	(1.40, 2.79)	<0.01

^a^ Violation of the PH assumption exist.

As could be seen from Figure 4B, 6 mm implants were at greater risk for failure than longer implants (HR = 3.53, *p* < 0.001). Immediate implantation (*n* = 1254) was a significant indicator for early implant failure, but the effect disappears after 10 years postsurgery (Figure 4C). Finally, implants combined with a GBR procedure were at greater risk for failure (HR = 1.85, *p* < 0.001) both in terms of early and late failures (Figure 4D). At patient level, heavy smokers and Diabetic patient are at a greater risk for experiencing a failure during implant service (Table 3).

**FIGURE 5:**
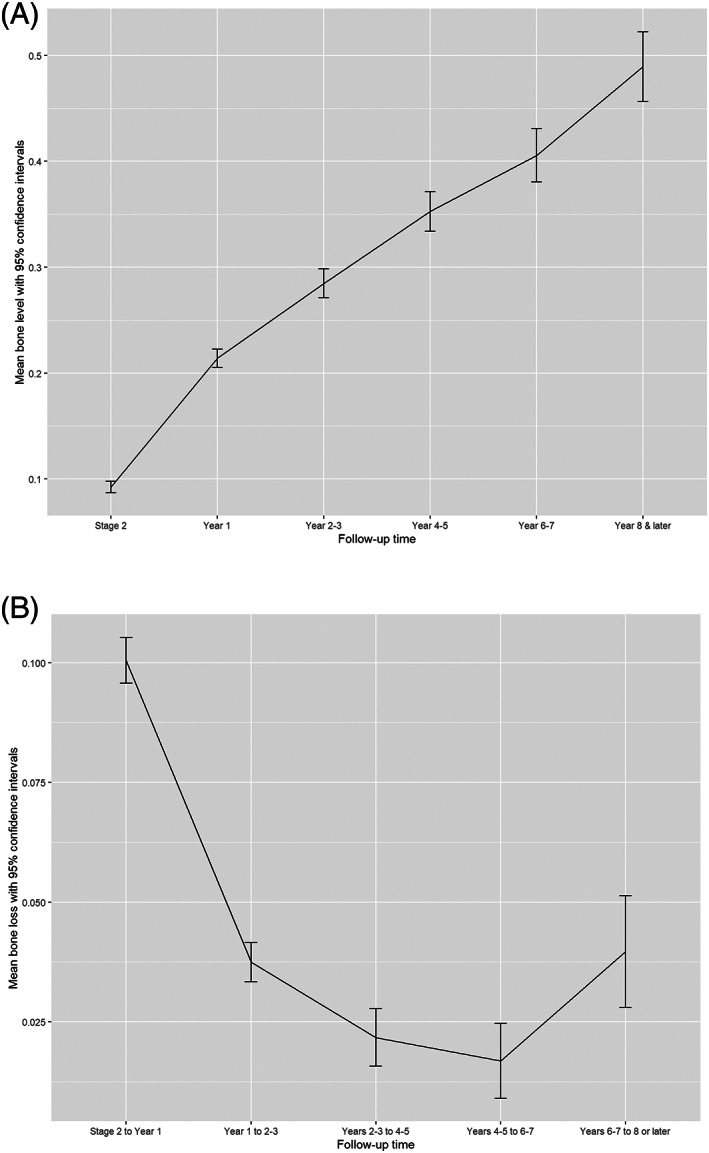
A, Mean Marginal bone level with 95% confidence interval (mm) by time. B, Mean Marginal bone loss with 95% Confidence interval over time (with upper 2% trimmer)

At stage two (*n* = 10 429) the mean bone level was 0.09 ± 0.28 mm while at 8–10 years (*n* = 1965) the mean bone level was 0.49 ± 0.74 mm (Figure 4). Bone loss following the first year was 0.05 ± 0.38 mm at years 2–3, and 0.21 ± 0.64 at year 8 or later. Throughout the study period, bone loss between two successive time points was prominent between stage 2 and year one. It slowed down until the fourth to fifth year. After that, bone loss was negligible and remained near to zero until 8–10 years of follow‐up, which again had greater amounts of bone loss (Figure 5).

**FIGURE 6:**
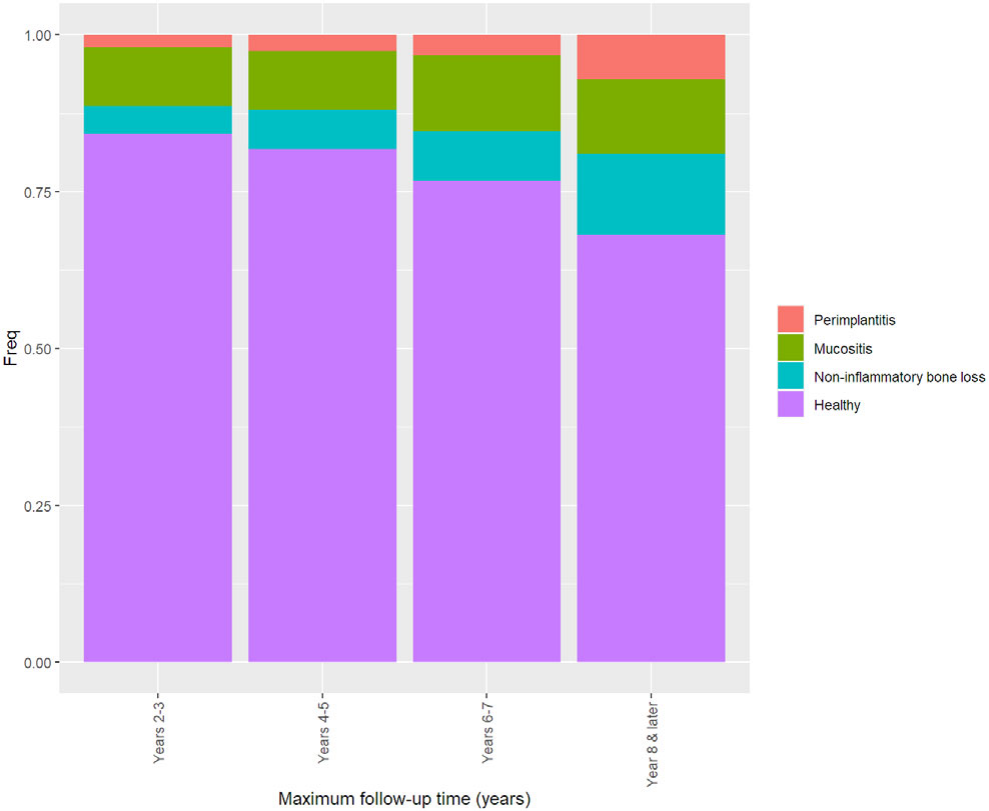
The incidence of peri‐implant mucositis and peri‐implantitis

The incidence of peri‐implant mucositis at the implant level was 9.4% at 2–3 years, 9.3% at 4–5 years, 12.1% at 6–7 years, and 11.9% at 8–10 years (Figure 6). The incidence of peri‐implantitis was 2%, 2.6%, 3.2%, and 7.1% at 2–3, 4–5, 6–7, and 8–10 years, respectively.

## 4 DISCUSSION

Implant therapy is regarded as a safe and reliable method of treating patients with complete or partial edentulism. The use of dental implants as a replacement for missing teeth has been increasing steadily, probably owing to the high predictability and survival rates, as reported in numerous studies.^4^, ^16^ Given the increasing popularity of dental implants, it is highly important to have a long‐term “real‐life” evaluation and analysis of this treatment performance.

The current study could serve as an example for a long‐term methodological data collection and analysis that can be performed in a clinical setting and contribute cumulatively to our base of knowledge in clinical practice. Despite the growing body of evidenced based knowledge, evidenced‐based practices are not always applied in the large‐scale environment of private practice clinical setting.^17^, ^18^ Implementation science intends to identify the barriers and present implementation strategies in an effort to enhance the uptake of these approaches. It is about trying to implement the knowledge we have into the daily practice ensuring our patients are receiving evidence‐based treatments. The employment of evidenced‐based research is required to provide optimal treatment for patients. Research is undeniably critical for patient care; however, we must be able to apply it and therefore, there is a need for more implementation science in dentistry.

Overall, the survival rates of the implants in the long‐term evaluation presented here, are within the reported rates in the literature both on the implant level and the patient level. It is important to emphasize though, that proper analysis with cumulative survival analysis is of utmost importance when reporting on long‐term results for such large cohorts.^19^ Setting proper expectations based on long‐term cumulative survival analysis is highly important when preparing patients for implant placement and receiving their informed consent. Patients should be well informed about the realistic survival and success rates of dental implants as well as the possible complications and morbidity.

When comparing implants in patients with multiple implants vs implants in patients who only have a single implant, the hazard ratio (HR) for time until failure, was 5.85 (*p* < 0.00001) which shows that patients with more implants present with higher chances for failure on the implant level. This might be attributed to the risk factors that are shared for tooth loss and implant failures such as periodontal disease, smoking and other systemic conditions.^20^

Immediate implantation was found to be a significant indicator for implant failure, but the effect disappears after 10 years postsurgery. This might imply that even though immediate implant placement should be considered as a risk factor for early implant failure, once the implant is stable and functioning for as long as 10 years following placement, it is no longer considered as a risk factor.

Finally, implants combined with a GBR procedure were at greater risk for failure (HR = 1.85, *p* < 0.001). It is highly crucial to realize that, as shown from the results of this work, implants in augmented bone are not as successful as implants placed in native bone and this risk presented not only in short term where infection may play a role but also longer term whereby the implant may be more at risk for peri‐implantitis or load related failures. This should be considered as part of the overall treatment plan and should be also shared with the patient.

Peri‐implant mucositis and peri‐implantitis are important entities that were observed in this current report. Long‐term evaluation and follow‐up for every implant patient are particularly important in order to identify early signs of these conditions. Early detection and proper intervention are crucial for favorable treatment outcomes.^21^

It is noteworthy, that the present report is based on data from a periodontal practice that puts strict emphasis on prevention, proper periodontal stabilization prior to implant placement as well as long‐term maintenance program and follow‐up. This might be one of the reasons for the rather predictable results shown. It is of utmost importance to highlight the role of proper preparation and maintenance for the long‐term outcomes.^19^ Retrospective studies, as their nature might present some risk of bias, which is a limitation of this study, however, studies like that are still important to assess risk factors over a long‐term follow‐up of a large number of patients and implants. Some of these limitations are related to confounding factors that cannot always be identified in retrospective analysis of cases. Multivariate analysis is an attempt to control some of the confounders but bias can still be present as part of the retrospective nature of this study.

Long‐term data from other practice‐based groups will enable comparison of the results and further analysis of confounding factors.

## 5 CONCLUSIONS

This study reported on long term follow‐up and analysis of success and survival of dental implants in a large cohort of patients treated in a periodontal clinic. Though the results are promising and encouraging in terms of survival and bone loss, it is important to emphasize the potential risk factors and consider them prior to dental implant placement.

## Data Availability

The data that support the findings of this study are available on request from the corresponding author. The data are not publicly available due to privacy or ethical restrictions.
